# An insight into pharmaceutical challenges with ionic liquids: where do we stand in transdermal delivery?

**DOI:** 10.3389/fbioe.2024.1454247

**Published:** 2024-08-06

**Authors:** Ankit Jain, Ashok K. Shakya, Shiv Kumar Prajapati, Mamdouh Eldesoqui, Nishi Mody, Sanjay K. Jain, Rajashri R. Naik, Umesh K. Patil

**Affiliations:** ^1^ Department of Pharmacy, Birla Institute of Technology and Science-Pilani, Pilani Campus, Pilani, India; ^2^ Pharmacological and Diagnostic Research Center, Faculty of Pharmacy, Al-Ahliyya Amman University, Amman, Jordan; ^3^ Institute of Pharmaceutical Research, GLA University, Mathura, India; ^4^ Department of Basic Medical Sciences, College of Medicine, AlMaarefa University, Riyadh, Saudi Arabia; ^5^ Department of Pharmaceutical Sciences, Dr. Harisingh Gour University, Sagar, India

**Keywords:** ionic liquid, biocompatibility, transdermal drug delivery, toxicity, challenges, sustainability, green cosolvents, ionic liquids drug delivery

## Abstract

Ionic liquids (ILs) represent an exciting and promising solution for advancing drug delivery platforms. Their unique properties, including broad chemical diversity, adaptable structures, and exceptional thermal stability, make them ideal candidates for overcoming challenges in transdermal drug delivery. Despite encountering obstacles such as side reactions, impurity effects, biocompatibility concerns, and stability issues, ILs offer substantial potential in enhancing drug solubility, navigating physiological barriers, and improving particle stability. To propel the use of IL-based drug delivery in pharmaceutical innovation, it is imperative to devise new strategies and solvents that can amplify drug effectiveness, facilitate drug delivery to cells at the molecular level, and ensure compatibility with the human body. This review introduces innovative methods to effectively address the challenges associated with transdermal drug delivery, presenting progressive approaches to significantly improve the efficacy of this drug delivery system.

## 1 Introduction

ILs have shown significant potential in drug delivery, particularly for enhancing transdermal delivery. Their ionic composition influences their ability to increase skin permeability. ILs are considered environmentally friendly, stable, non-volatile, and non-flammable, making them preferable to conventional solvents. Because of their unique and customizable physical, chemical, and biological properties, ILs are valuable as cosolvents and materials in drug delivery systems and active pharmaceutical ingredient (API) formulations. ILs can be customized in formulations to enhance solubility and stability, thus improving drug bioavailability. Their properties enable the development of innovative delivery methods and can potentially increase treatments’ effectiveness and safety. Furthermore, the, non-toxic, and biodegradable nature of certain ILs underscores their suitability for pharmaceutical use, ensuring minimal environmental impact and better patient compliance. As a result, ILs show great potential for the advancement of drug delivery systems (Lu et al., 2022). ILs can create nanoparticles and micelles, which can deliver therapeutic agents to specific areas of the body. This method increases the effectiveness of drugs and reduces potential side effects. Additionally, ILs can be utilized to produce solid dispersions, significantly improving the solubility and bioavailability of poorly water-soluble drugs (Kapre et al., 2024). This improvement allows better drug absorption and distribution, ultimately leading to better therapeutic results. Consequently, ILs play a vital role in developing new pharmaceutical products, contributing to the creation of treatments that are not only more effective but also safer for patients (Moshikur et al., 2021; Shukla et al., 2023).

**FIGURE 1:**
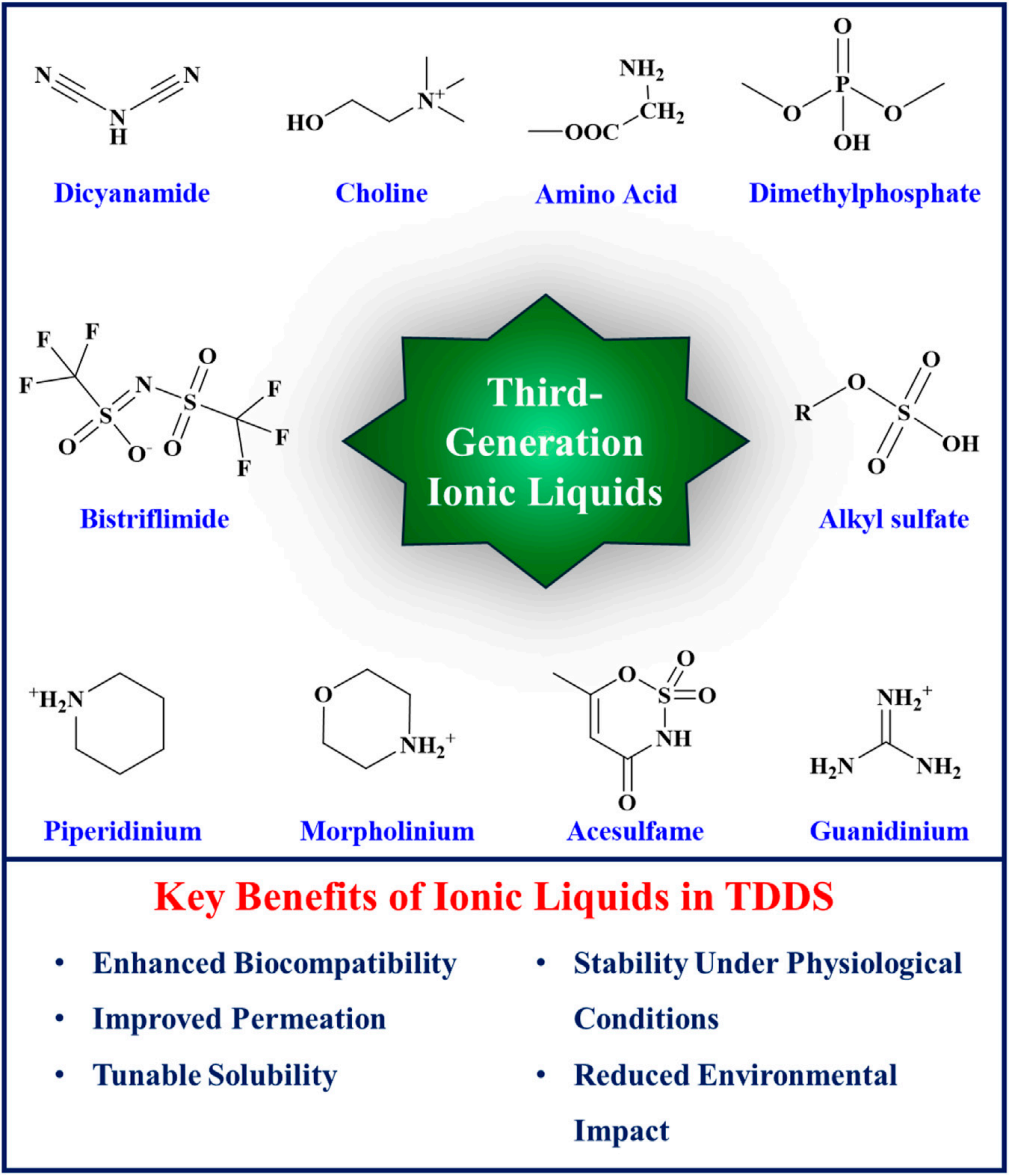
Third Generation Ionic Liquids and their Key Benefits.

ILs have been categorized into three generations based on their discovery timeline and evolving properties. The development of ILs has progressed from the first to the third generation, each characterized by distinct chemical structures and applications. The first generation of ILs, primarily utilized in electroplating, involved the combination of dialkyl imidazolium and alkylpyridinium cations with metal halide anions. These ILs exhibited unique physical properties such as high thermal stability, low melting points, and broad liquid ranges, making them suitable substitutes for certain organic solvents. However, they were hindered by low biodegradability, high aquatic toxicity, and high production costs. The second generation of ILs, which are stable in both water and air, was synthesized using cations such as dialkyl imidazolium, alkylpyridinium, ammonium, and phosphonium, and anions such as tetrafluoroborate and hexafluorophosphate (Brahma et al., 2024). These ILs presented customizable physical and chemical properties, including melting point, viscosity, thermal stability, hydrophilicity, solubility, toxicity, and biodegradability, accomplished by fine-tuning and modifying their anions, cations, and substituents (Magina et al., 2021). Second-generation ILs in drug delivery systems face significant challenges, including toxicity, biocompatibility issues, regulatory barriers, and environmental concerns (Moshikur et al., 2020; Shukla et al., 2023). Formulation and compatibility complexities also limit their use (Amaral et al., 2021). The third generation of ILs introduced natural sources for anions (e.g., amino acids, fatty acids) and cations (e.g., choline). These ILs maintained promising physical and chemical properties and provided reduced toxicity and enhanced biodegradability. The breakthrough of third-generation ILs has prompted increasing research into their applications in biomedicine, including transdermal drug delivery systems, due to their improved safety profiles and environmental compatibility. Figure 1 illustrates the structures and key benefits of third-generation of ionic liquids.

**FIGURE 2:**
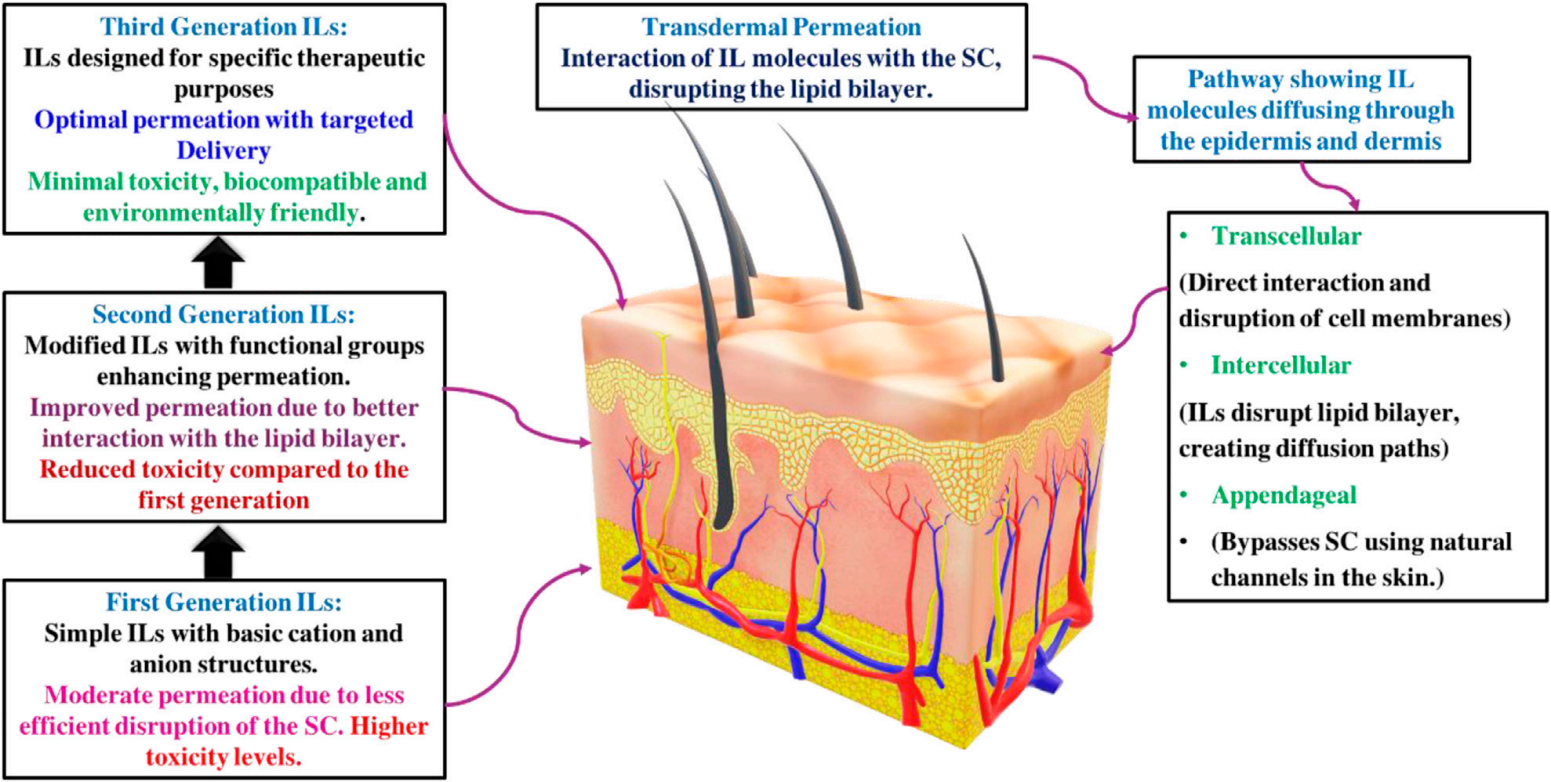
Potential application of ILs in transdermal delivery.

The role of ILs as chemical permeation enhancers is well-documented. ILs have been shown to effectively facilitate transcellular and paracellular transport, bypassing the stratum corneum (SC) by disrupting cell integrity, fluidizing lipids, creating diffusional pathways, and extracting lipid components from the SC. These properties of ILs undoubtedly promise to improve efficiency in transdermal drug delivery, leading to more effective treatments and better patient outcomes (Egorova et al., 2017; Kundu et al., 2017). Room-temperature ionic liquids (RTILs) are highly adaptable for various applications, mainly as chemical permeation enhancers in transdermal drug delivery. They enhance the transport of substances through cells and cellular barriers by disrupting cellular integrity, fluidizing lipids, creating diffusional pathways, and extracting lipids from the outermost layer. This remarkable adaptability significantly improves the performance of transdermal drug delivery systems, resulting in better clinical outcomes (Sidat et al., 2019). Moshikur et al. conducted an in-depth review of strategies for designing and developing ILs, with particular emphasis on biocompatible IL-based formulations and delivery systems. They highlighted the applications and advantages of ILs over traditional organic solvents in drug formulations and delivery mechanisms (Moshikur et al., 2023). Sidat et al. (2019) conducted a detailed review of the potential of biodegradable ILs to advance TDDS. They discussed ILs’ roles as permeation enhancers, drug modification agents, and active pharmaceutical ingredients, suggesting their synergistic use with other chemical permeation enhancers. Additionally, the review addressed the challenges associated with ILs in TDDS, such as the physical properties of ILs, Interactions of ILs with Biomolecules and Membranes, and the impact of permeation enhancement. Shukla et al. (2023) reviewed the expanding role of ILs in drug delivery systems, emphasizing their potential to enhance the solubility of poorly soluble drugs. They highlighted ILs’ customizable properties and recent advancements, showcasing their growing importance in pharmaceutical and biomedical applications. In another literature, Ali et al. (2022) explored ILs for drug delivery and highlighted their tunable properties that address issues with traditional permeation enhancers. They focused on biocompatible IL-based systems using advanced cations and anions to improve permeability and reduce cytotoxicity and skin irritation, showcasing recent advancements and promising applications in drug delivery. This mini-review differs from the aforementioned literature by specifically addressing the challenges related to the use of ILs in TDDS and highlighting innovative approaches to address these challenges. This also focuses on integrating ILs with novel drug delivery carriers to improve their applicability and increase therapeutic potential via the transdermal route. This approach aims to provide a comprehensive understanding of the advances in this field while filling existing gaps and suggesting future directions for research and development in IL-based transdermal drug delivery systems. Figure 2 illustrates the potential application of ILs in transdermal delivery.

## 2 Challenges associated with ionic liquids

### 2.1 Skin permeability

**FIGURE 3:**
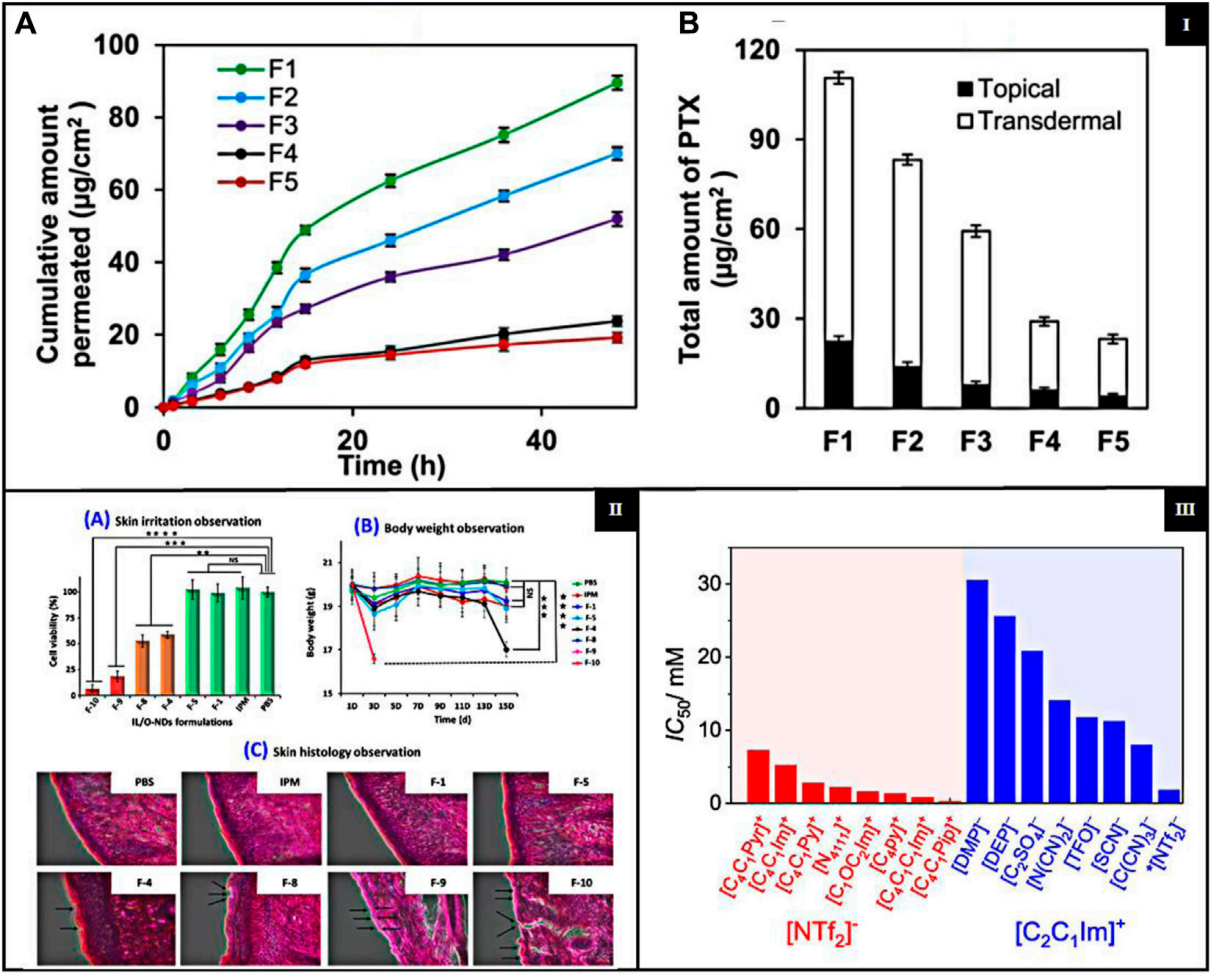
**[I] (A)** Transdermal permeation profiles of PTX delivered by various MFs with different SAIL/co-surfactant ratios (S/Co); **(B)** total PTX delivery by various MFs with different S/Co. *{adapted with permission from Ali et al.* (Ali et al., 2021)*. Copyright {2021} American Chemical Society.}* **[II] (A)** Cell viability on the human LabCyte EPI-MODEL. **(B)** Body weight of mouse during the *in vivo* biocompatibility study and **(C)** skin histopathological experiment *{adapted with permission from Uddin et al.* (Uddin et al., 2021)*. Copyright {2021} American Chemical Society}*; **[III]** Cytotoxicity for ILs with anion and different cations (red left side) and ILs with cation and various anions (blue right side) *(*adapted *from Musial et al.* (Musiał et al., 2021)*, CC-BY 4.0 license).*

The skin permeability of ILs is mainly affected by their physical and chemical properties, which in turn depend on the structures. However, the transdermal route of drug delivery is challenged by the impenetrable nature of SC; hence, achieving optimum permeability remains an issue (Paz Ramos et al., 2020). Significant challenges in using ILs to improve skin permeability include possible skin irritation and toxicity due to their disruptive effects on the skin barrier (Leitch et al., 2020); the cation predominantly influences the skin irritability of ILs; however, the anion also contributes to this effect (Patil and Sasai, 2008). ILs containing alicyclic cations and ammonium cations, such as morpholinium and pyrrolidinium, exhibit lower toxicity and skin irritability than those based on imidazolium and pyridinium cations (Zhang et al., 2006). The variability in efficacy and safety among different ILs requires extensive screening and optimization, which is resource-intensive. Ensuring that ILs are nontoxic, biodegradable, and environmentally friendly while maintaining efficacy is complex. Additionally, the stability of the formulation and compatibility with other pharmaceutical ingredients add to the challenges of developing effective transdermal delivery systems. To overcome the challenge of transdermal delivery of paclitaxel (PTX) caused by its low water solubility, a micelle formulation (MF) was developed using biocompatible surfactants, cholinium oleate ([Cho][Ole]) and sorbitan monolaurate (Span-20). This formulation effectively improved the solubility and stability. Stable micelles formed significantly increased the absorption of PTX. According to Figure 3IA, the transdermal permeation profiles of various formulations showed the superior performance of SAIL-based MF in delivering higher rates of PTX compared to other carriers. Figure 3IB illustrates the total PTX delivery from different formulations over 48 h, with the SAIL-based MF showing a markedly higher delivery efficiency, which confirms its effectiveness in enhancing the transdermal permeation of PTX (Ali et al., 2021). Another study reported ILs as TDD for the treatment of rheumatoid arthritis. At the same time, another study introduced a new transdermal IL patch, SIHDD-PSA, utilizing semi-ionic hydrogen bonding to enhance the delivery of Actarit and Ketoprofen for rheumatoid arthritis treatment. By forming a complex with triethylamine, the ILs improved the solubility and permeability, significantly increasing Actarit loading by approximately 11.34 times and enhancing *in vitro* permeability by 5.46 times, with similar improvements for Ketoprofen, i.e., 2.39 times increase in permeability. This approach addresses the challenge of skin barrier permeation. It mitigates gastrointestinal side effects associated with oral drug delivery, demonstrating a promising advancement in transdermal therapeutic systems through *in vitro* and *in vivo* evaluations (Zhang et al., 2024).

### 2.2 Biocompatibility and biodegradation

ILs are widely used in pharmaceuticals and require long-term biocompatibility and safety for patients. First-generation ILs have attracted attention for their unique physical properties; They are sensitive to water and air and have poor biodegradability (Zhuo et al., 2024). ILs can have biocompatibility issues, including toxicity and low biodegradability. These issues can pose challenges to their safety in various applications. However, biocompatible ILs (Bio-ILs) have been developed as a greener alternative to conventional ILs and organic/inorganic solvents (Kanjilal et al., 2023; Zhuo et al., 2024). The biodegradability of ILs is determined by their molecular structure, including anions, cations, and functional groups, and is typically measured concerning these components (Elmobarak et al., 2023). A study investigates lipid-based ionic liquids (LBILs) as carriers for TDDS and demonstrated their biocompatibility. The LBIL-based formulations, especially those using [EDMPC] [linoleate]/O-NDs, showed excellent biocompatibility in both *in vitro* and *in vivo* cytotoxicity studies. These formulations did not cause a significant reduction in cell viability or adverse skin reactions Figure 3IIA, B, C, suggesting that they are safe for pharmaceutical use. The results highlight the potential of LBILs as promising biocompatible carriers to improve the transdermal delivery of the peptide drug (Uddin et al., 2021). Another study of biocompatible ILs addresses challenges such as low solubility and poor skin penetration of therapeutic agents. By incorporating surface-active ILs, the study improved the skin permeation and stability of antigenic proteins and adjuvants, demonstrating improved biocompatibility. Preclinical studies showed that the IL-assisted delivery systems (ILDS) were nonirritant and safe for topical applications. ILDS significantly enhanced antigenic protein delivery and immunogenic response, making them promising candidates for transdermal drug delivery and immunotherapy applications (Chowdhury et al., 2021).

### 2.3 Impact of impurities

Impurities in ILs are crucial, especially their influence on properties such as viscosity. Even minor impurities such as water and halides can significantly alter the properties of the IL. However, less attention has been paid to the third generation of biocompatible ILs to trace contaminants and their biological interactions (Le Donne and Bodo, 2021). Accurate characterization and quantification of contaminants are critical as they affect material properties and biological safety. Techniques such as Karl Fischer titration, HPLC, and Volhard titration (for halides) are used to measure impurities. Improved synthetic methods can reduce impurities by using precursors that yield easily removable by-products, such as water and carbon dioxide, resulting in pure ILs (Stark et al., 2008; Curreri et al., 2021). The impact of impurities on hydrophilic ILs used in gel-based dermal formulations emphasizes that contaminants such as organic materials, halides, and protic residues from incomplete reactions significantly influence the properties and effectiveness. These impurities can drastically alter pH levels, reduce antimicrobial activity, and affect formulation stability by changing viscosity and phase behavior. Such changes are crucial because they can compromise the delivery efficiency and safety of drug substances in dermal applications, emphasizing the need for rigorous control over the purity of IL in pharmaceutical formulations for TDD (Dobler et al., 2016).

### 2.4 Toxicity

Toxicity is a crucial factor in evaluating ILs, as earlier generations had high toxicity, making them unsuitable for medical applications. Furthermore, the structural properties of the cations in the ILs influence their toxicity. ILs containing cations such as ammonium, imidazolium, morpholinium, piperidinium, and pyrrolidinium are often associated with high toxicity and reduced degradability (Mano et al., 2020; Miao et al., 2022). The presence of hydrophilic anions such as chloride, bromide, and organic groups such as carboxylates and sulfonates reduces the overall toxicity of IL, indicating a significant impact on their safety (Flieger and Flieger, 2020; Cho et al., 2021; Chen et al., 2024). Including a polar functional group within the alkyl chain has been shown to reduce both the toxicity and the biodegradation efficiency of ILs. This suggests the possibility of modifying ILs by integrating particular functional groups into their structure, resulting in a more eco-friendly product (Navti et al., 2022). As the lipophilicity or length of the alkyl chain increases, there is an increase in both degradation rate and toxicity, furthermore, from the perspectives of toxicology and microbial degradation (Pham et al., 2010). ILs with polar ether, hydroxyl, and nitrile groups on their side chains show lower cytotoxicity than those with essential alkyl chains (Thuy et al., 2010). The concept of ILs as green chemicals is based on their non-volatile and non-flammable nature, which distinguishes them from conventional organic solvents. Replacing volatile organic solvents with non-volatile ILs in chemical and biological processes can mitigate the risks of inhalation exposure, flammability, and environmental contamination. However, more extensive studies on toxicity and biocompatibility are required to confirm that ILs are intrinsically safe and environmentally friendly. Research shows numerous ILs pose risks to various biological entities, from nucleic acids to multicellular organisms (Agatemor et al., 2018; Shukla et al., 2023). Bioinspired ILs have been formulated to exhibit improved biodegradability and a lower toxicity profile to address this challenge. Among these, choline-based ILs are the most extensively researched (Agatemor et al., 2018; Sidat et al., 2019). A study evaluated the cytotoxicity of 31 ILs in human dermal fibroblasts. The results showed that imidazolium-based ILs with dialkyl phosphate or ethyl sulfate anions had the lowest cytotoxicity. In particular, 1,3-diethyl imidazolium ethyl sulfate is promising as a hydraulic fluid, like the commercial 1-ethyl-3-methylimidazolium ethyl sulfate. Dialkyl phosphate-based ILs proved to be effective as solvents for lignocellulose biomass and eco-friendly bioplastic extractants. Moreover, pyrrolidinium and cyano-based ILs, used in heat transfer and nanofluids, also exhibited lower toxicity compared to other ILs. The NHDF cell line was used to assess IC_50_ values of ILs, with findings presented in Figure 3III (Musiał et al., 2021). A recent study investigates the safety and suitability for the biological use of newly created double-charged ILs. These ILs, including ibuprofen and piroxicam, are specifically formulated to improve the dissolution of drugs with low solubility. These ILs, which are composed of carboxylic anions and ammonium cations, significantly increased the solubility of drugs in both water and phosphate buffer solutions. This indicates promising potential for use in drug delivery systems. The evaluation of cytotoxicity using fibroblast L929 cells showed that most of these ILs had low toxicity, with IC_50_ values higher than 100 mM, demonstrating good biocompatibility (Agostinho et al., 2021).

### 2.5 Stability issues

Stability is a prerequisite for the use of ILs. ILs can be used at elevated temperatures, as electrolytes, or under irradiation. Therefore, the thermal, electrochemical, and radiolytic stabilities of ILs are essential and need to be known before their use. The decomposition temperature of ILs, which ranges from 200°C to 400°C, is strongly influenced by the type of anion, highlighting its crucial role in thermal stability. In addition to structural factors such as the type and modification, this stability is also related to the coordination ability of the anion, its nucleophilicity, and hydrophilicity, alongside structural factors like cation type and alterations of the cation (Cao and Mu, 2014). Although ILs are generally non-flammable, they are susceptible to thermal decomposition. When exposed to high temperatures during processes, these changes can cause ILs to degrade structurally, potentially producing undesirable by-products and leading to accidents (He et al., 2021).

In our discussion, we have explored the various complexities associated with IL-based transdermal delivery. However, a range of emerging advancements are specifically designed to address these challenges. These cutting-edge approaches are summarized in detail in Table 1.

**TABLE 1:** Innovative approaches to tackle challenges with ILs-based transdermal delivery.

Role of ILs	Composition of ILs	Drug	Remarks	Ref**.**
Transdermal delivery enhancer	1-hydroxyethyl-3-methylimidazolium chloride ([HOEIM]Cl) and 1-butyl-3-methylimidazolium dodecane sulfate ([BMIM]C12SO3)	Dencichine	IL-based microemulsions improved Dencichine solubility and permeation, showing significant hemostatic activity	Wang et al. (2018)
Transdermal delivery enhancer	Choline formate, choline lactate, choline propionate	Acyclovir	IL-based microemulsions improved acyclovir solubility and skin permeation, resulting in a significant improvement in drug delivery rates	Islam et al. (2020a)
Transdermal delivery enhancer	Choline and Geranic Acid (CAGE)	Insulin	The CAGE variants were purposefully designed and applied in *ex vivo* porcine skin studies, resulting in significantly elevated insulin delivery rates to the dermis. This notable improvement was directly attributed to the inherent ability of CAGE’s to disrupt the lipid structure of the stratum corneum, ultimately leading to increased skin permeability	Tanner et al. (2018)
Enhancing Solubility, Permeability, and Stability	1-hexyl-3-methylimidazolium chloride [Hmim] [Cl] and 1-butyl-3-methylimidazolium hexafluorophosphate [Bmim][PF6]	Piroxicam	ILs significantly improved piroxicam permeability in nanoemulsions by forming a stable micelle structure that disrupts the skin’s barrier. This enhancement led to a 93% increase in drug release when using [Hmim][Cl]. The nanoemulsions exhibited good physical stability over extended storage periods, maintaining homogeneity and droplet size. Improved stability was observed when using a surfactant ratio of 2:1	Mahamat Nor et al. (2017)
Enhancing Solubility, Permeability, and Stability	Acyclovir Methotrexate	Ammonium acetate ILs ([EMIM][OAc], [TEA][OAc], [DEA][OAc])	ILs of ammonium acetate in oil-in-water microemulsions significantly increased the solubility and permeability. Stability testing showed improved drug encapsulation and reduced particle size over time, indicating a stable and effective delivery system	Kandasamy et al. (2018)
Enhancing solubility, permeability, and impurity analysis	Caffeine, Testosterone	Hexylpyridinium chloride [HPyr][Cl], Choline dihydrogen phosphate [CDHP], 1-ethyl-3-methylimidazolium ethyl sulfate [EMIM][EtSO_4_]	ILs increase the solubility and skin penetration of caffeine and testosterone. [HPyr] [Cl] significantly boosted caffeine uptake and showed solid antimicrobial activity. The formulations were stable for 3 months, but impurities affected pH values, especially in [EMIM][EtSO4]	Dobler et al. (2016)
Enhancing solubility, permeability	Donepezil	Dicarboxylic ILs (various)	The dicarboxylic IL forms of donepezil significantly increased solubility by more than 100 times in water and PBS compared to the free base. There was also a noticeable improvement in skin permeability, with up to 13 times higher permeation. Stability studies did not indicate significant degradation. Impurities can be removed by evaporating in a dry oven	Dinh et al. (2022)
Enhancing Solubility, Permeability, and Biocompatibility Study	Acyclovir	Choline as the cation, and an AA (glycine, alanine, or serine) as the anion	Enhanced permeation due to reduced skin barrier function; high cell viability (>85%); no significant skin irritation; biocompatible and toxicity reduced compared to imidazolium-based ILs	Islam et al. (2020b)
Solubilizing agent and permeation enhancer	Antigen peptide	choline−fatty acids ([Cho][FA])	[Cho] [C18:1] facilitated the dissolution of a hydrophilic peptide in an oil-based skin penetration enhancer, significantly improving transdermal delivery. The formulation caused minor skin irritation and showed lower cytotoxicity than other ILs	Islam et al. (2021)
Enhancing stability and biocompatibility	Acyclovir	ILs of choline propionate ([Ch][Pro]) and choline oleate ([Ch][Ole]) surface-active ILs (SAILs)	IL-based microemulsions significantly improved acyclovir solubility and skin permeation. The IL/O patch maintained stable drug content and physical appearance for 30 days at room temperature, with minimal skin irritation and high biocompatibility	Islam et al. (2024)
Enhancing stability and permeability	Insulin	LBIL based on DMPC (1,2-dimyristoyl-sn-glycero-3-ethyl-phosphatidylcholine (EDMPC) with linoleic acid (Lin))	The use of IL-mediated ethosomes led to significant improvements in insulin solubility and skin permeation. These ethosomes demonstrated long-term stability at 4°C and 25°C for over 12 weeks. They also exhibited high encapsulation efficiency (98%–99%), good biocompatibility and low cytotoxicity. Overall, they effectively addressed the challenges associated with transdermal delivery of high-molecular-weight drugs by enhancing stability and penetration	Nabila et al. (2024)
Enhancing permeability and biocompatibility	5-Fluorouracil (5-FU)	Choline Alanine ([Cho][Ala]), Choline Proline ([Cho][Pro]) with Oleic Acid (OA)	IL-based hydrogels significantly improved the solubility and skin permeation of 5-FU by 255 times ([Cho][Ala]) and 250 times ([Cho][Pro]) compared to PBS. These hydrogels exhibited excellent mechanical properties, biocompatibility, and high cell viability (>92%) in standard and cancerous cell lines. Therefore, they are considered promising candidates for the treatment of breast cancer	Pansuriya et al. (2024)
Enhanced Stability and controlled release	Isoniazid	[2-(methacryloyl-oxy)-ethyl]trimethyl-ammonium chloride (TMAMA_Cl‾) with or without *p*-amino salicylic acid (PAS)	IL-based matrices polymerized with PAS and employed as a dual drug delivery system. The amphiphilic nature of these copolymers favors encapsulating and releasing encapsulated drugs. Cytotoxic evaluation using the MTT test confirms the non-toxic nature of these systems on human non-tumorigenic lung epithelial cell line (BEAS-2B) cell lines	Niesyto et al. (2024a)
Enhanced Stability and controlled release	**Piperacillin/Tazobactam**	[2-(methacryloyloxy)ethyl]-trimethylammonium chloride (TMAMA) ILs	Graft copolymers based on [2-(methacryloyloxy)-ethyl]-trimethyl-ammonium chloride (TMAMA) units were utilized as dual-drug carriers with encapsulated tazobactam and ionically conjugated piperacillin for co-delivery applications	Niesyto et al. (2024b)

## 3 Conclusion and future perspectives

ILs represent an exciting area of research in targeted drug delivery because of their remarkable structural versatility and accessible coupling properties. The ability to modify IL properties for various biological applications underscores their potential to enhance drug permeability across physiological barriers, increase the solubility of poorly soluble drugs, and develop innovative IL-based drug delivery formulations. The effectiveness of ILs as solvent media, solubility or permeation enhancers, and potential drug delivery carriers has sparked significant interest in drug delivery. Moreover, the integration of ILs with nanostructures has marked a significant advancement. The combination of ILs with nanoparticles not only enhances stability, activity, and performance and enables precise control over size and morphology. This synergy is valuable in drug delivery applications and has the potential to lead to superior therapeutic outcomes. The development of combined IL-nanostructures drug-delivery systems holds the promise of expanding pharmaceutical applications and improving therapeutic efficiency. Future research must prioritize critical areas to unleash the potential of ILs in drug delivery. Additional efforts are crucial in fine-tuning the properties of ILs to maximize their efficacy as drug delivery systems. This involves exploring new IL compositions and combinations to enhance their biocompatibility and therapeutic effectiveness. Additionally, comprehensive investigations of the safety and toxicity of ILs to address potential side effects. Understanding the long-term impact of IL exposure on human health will be pivotal to their clinical implementation. The transition of IL-based drug delivery systems from research to clinical use will be facilitated by formulating precise regulatory guidelines for their approval. Thorough clinical trials will be indispensable to confirm IL-based formulations’ safety and therapeutic benefits for extensive use in medical practice. Additionally, further research is vital to combine ILs with nanotechnology to leverage the synergistic benefits fully. This includes developing innovative IL-nanotechnology structures tailored to specific therapeutic applications. By addressing these areas, the field of IL-based drug delivery can make strides in developing highly efficient, safe, and therapeutic options that have the potential to revolutionize drug delivery and improve patient outcomes.

## References

[B1] Agatemor C. Ibsen K. N. Tanner E. E. L. Mitragotri S. (2018). Ionic liquids for addressing unmet needs in healthcare. Bioeng. Transl. Med. 3 (1), 7–25. 10.1002/btm2.10083 29376130 PMC5773981

[B2] Agostinho D. A. Santos F. Esperança J. M. Duarte A. R. Reis P. M. (2021). New non-toxic biocompatible dianionic ionic liquids that enhance the solubility of oral drugs from BCS class II. J. Ionic Liq. 1 (1), 100003. 10.1016/j.jil.2021.100003

[B3] Ali M. K. Moshikur R. M. Goto M. Moniruzzaman M. (2022). Recent developments in ionic liquid-assisted topical and transdermal drug delivery. Pharm. Res. 39 (10), 2335–2351. 10.1007/s11095-022-03322-x 35773446

[B4] Ali M. K. Moshikur R. M. Wakabayashi R. Moniruzzaman M. Goto M. (2021). Biocompatible ionic liquid-mediated micelles for enhanced transdermal delivery of paclitaxel. ACS Appl. Mater. Interfaces 13 (17), 19745–19755. 10.1021/acsami.1c03111 33891816

[B5] Amaral M. Pereiro A. B. Gaspar M. M. Reis C. P. (2021). Recent advances in ionic liquids and nanotechnology for drug delivery. Nanomedicine 16 (1), 63–80. 10.2217/nnm-2020-0340 33356551

[B6] Brahma S. Gardas R. L. (2024). “History and development of ionic liquids,” in Handbook of ionic liquids: fundamentals, applications, and sustainability. Editors Rajkhowa S. Singh P. Sen A. Sarma J. (John Wiley and Sons), 1–28.

[B7] Cao Y. Mu T. (2014). Comprehensive investigation on the thermal stability of 66 ionic liquids by thermogravimetric analysis. Industrial Eng. Chem. Res. 53 (20), 8651–8664. 10.1021/ie5009597

[B8] Chen X. Li Z. Yang C. Yang D. (2024). Ionic liquids as the effective technology for enhancing transdermal drug delivery: design principles, roles, mechanisms, and future challenges. Asian J. Pharm. Sci. 19, 100900. 10.1016/j.ajps.2024.100900 38590797 PMC10999516

[B9] Cho C.-W. Pham T. P. T. Zhao Y. Stolte S. Yun Y.-S. (2021). Review of the toxic effects of ionic liquids. Sci. Total Environ. 786, 147309. 10.1016/j.scitotenv.2021.147309 33975102

[B10] Chowdhury M. R. Moshikur R. M. Wakabayashi R. Moniruzzaman M. Goto M. (2021). Biocompatible ionic liquids assisted transdermal co-delivery of antigenic protein and adjuvant for cancer immunotherapy. Int. J. Pharm. 601, 120582. 10.1016/j.ijpharm.2021.120582 33872711

[B11] Curreri A. M. Mitragotri S. Tanner E. E. (2021). Recent advances in ionic liquids in biomedicine. Adv. Sci. 8 (17), 2004819. 10.1002/advs.202004819 PMC8425867 34245140

[B12] Dinh L. Lee S. Abuzar S. M. Park H. Hwang S.-J. (2022). Formulation, preparation, characterization, and evaluation of dicarboxylic ionic liquid donepezil transdermal patches. Pharmaceutics 14 (1), 205. 10.3390/pharmaceutics14010205 35057101 PMC8812279

[B13] Dobler D. Schmidts T. Zinecker C. Schlupp P. Schäfer J. Runkel F. (2016). Hydrophilic ionic liquids as ingredients of gel-based dermal formulations. Aaps Pharmscitech 17, 923–931. 10.1208/s12249-015-0421-y 27435197

[B14] Egorova K. S. Gordeev E. G. Ananikov V. P. (2017). Biological activity of ionic liquids and their application in pharmaceutics and medicine. Chem. Rev. 117 (10), 7132–7189. 10.1021/acs.chemrev.6b00562 28125212

[B15] Elmobarak W. F. Almomani F. Tawalbeh M. Al-Othman A. Martis R. Rasool K. (2023). Current status of CO2 capture with ionic liquids: development and progress. Fuel 344, 128102. 10.1016/j.fuel.2023.128102

[B16] Flieger J. Flieger M. (2020). Ionic liquids toxicity—benefits and threats. Int. J. Mol. Sci. 21 (17), 6267. 10.3390/ijms21176267 32872533 PMC7504185

[B17] He H. Pan Y. Meng J. Li Y. Zhong J. Duan W. (2021). Predicting thermal decomposition temperature of binary imidazolium ionic liquid mixtures from molecular structures. ACS omega 6 (20), 13116–13123. 10.1021/acsomega.1c00846 34056461 PMC8158806

[B18] Islam M. R. Chowdhury M. R. Wakabayashi R. Kamiya N. Moniruzzaman M. Goto M. (2020a). Ionic liquid-in-oil microemulsions prepared with biocompatible choline carboxylic acids for improving the transdermal delivery of a sparingly soluble drug. Pharmaceutics 12 (4), 392. 10.3390/pharmaceutics12040392 32344768 PMC7238071

[B19] Islam M. R. Chowdhury M. R. Wakabayashi R. Tahara Y. Kamiya N. Moniruzzaman M. (2020b). Choline and amino acid based biocompatible ionic liquid mediated transdermal delivery of the sparingly soluble drug acyclovir. Int. J. Pharm. 582, 119335. 10.1016/j.ijpharm.2020.119335 32311469

[B20] Islam M. R. Uddin S. Chowdhury M. R. Wakabayashi R. Moniruzzaman M. Goto M. (2021). Insulin transdermal delivery system for diabetes treatment using a biocompatible ionic liquid-based microemulsion. ACS Appl. Mater. Interfaces 13 (36), 42461–42472. 10.1021/acsami.1c11533 34460218

[B21] Islam R. Nabila F. H. Wakabayashi R. Kamiya N. Moniruzzaman M. Goto M. (2024). Ionic Liquid-Based patch formulation for enhanced transdermal delivery of sparingly soluble drug. J. Mol. Liq. 397, 124184. 10.1016/j.molliq.2024.124184

[B22] Kandasamy S. Moniruzzaman M. Sivapragasam M. Shamsuddin M. R. Mutalib M. I. A. (2018). Formulation and characterization of acetate based ionic liquid in oil microemulsion as a carrier for acyclovir and methotrexate. Sep. Purif. Technol. 196, 149–156. 10.1016/j.seppur.2017.08.044

[B23] Kanjilal B. Zhu Y. Krishnadoss V. Unagolla J. M. Saemian P. Caci A. (2023). Bioionic liquids: enabling a paradigm shift toward advanced and smart biomedical applications. Adv. Intell. Syst. 5 (5), 2200306. 10.1002/aisy.202200306

[B24] Kapre S. Palakurthi S. S. Jain A. Palakurthi S. (2024). DES-igning the future of drug delivery: a journey from fundamentals to drug delivery applications. J. Mol. Liq. 400, 124517. 10.1016/j.molliq.2024.124517

[B25] Kundu N. Roy S. Mukherjee D. Maiti T. K. Sarkar N. (2017). Unveiling the interaction between fatty-acid-modified membrane and hydrophilic imidazolium-based ionic liquid: understanding the mechanism of ionic liquid cytotoxicity. J. Phys. Chem. B 121 (34), 8162–8170. 10.1021/acs.jpcb.7b06231 28756672

[B26] Le Donne A. Bodo E. (2021). Cholinium amino acid-based ionic liquids. Biophys. Rev. 13 (1), 147–160. 10.1007/s12551-021-00782-0 33747249 PMC7930144

[B27] Leitch A. C. Abdelghany T. M. Probert P. M. Dunn M. P. Meyer S. K. Palmer J. M. (2020). The toxicity of the methylimidazolium ionic liquids, with a focus on M8OI and hepatic effects. Food Chem. Toxicol. 136, 111069. 10.1016/j.fct.2019.111069 31883992 PMC6996134

[B28] Lu B. Liu T. Wang H. Wu C. Chen H. Liu Z. (2022). Ionic liquid transdermal delivery system: progress, prospects, and challenges. J. Mol. Liq. 351, 118643. 10.1016/j.molliq.2022.118643

[B29] Magina S. Barros-Timmons A. Ventura S. P. Evtuguin D. V. (2021). Evaluating the hazardous impact of ionic liquids–challenges and opportunities. J. Hazard. Mater. 412, 125215. 10.1016/j.jhazmat.2021.125215 33951860

[B30] Mahamat Nor S. B. Woi P. M. Ng S. H. (2017). Characterisation of ionic liquids nanoemulsion loaded with piroxicam for drug delivery system. J. Mol. Liq. 234, 30–39. 10.1016/j.molliq.2017.03.042

[B31] Mano B. Jesus F. Gonçalves F. J. Ventura S. P. Pereira J. L. (2020). Applicability of heuristic rules defining structure–ecotoxicity relationships of ionic liquids: an integrative assessment using species sensitivity distributions (SSD). Green Chem. 22 (18), 6176–6186. 10.1039/d0gc02486d

[B32] Miao S. Atkin R. Warr G. (2022). Design and applications of biocompatible choline amino acid ionic liquids. Green Chem. 24 (19), 7281–7304. 10.1039/d2gc02282f

[B33] Moshikur R. M. Carrier R. L. Moniruzzaman M. Goto M. (2023). Recent advances in biocompatible ionic liquids in drug formulation and delivery. Pharmaceutics 15 (4), 1179. 10.3390/pharmaceutics15041179 37111664 PMC10145603

[B34] Moshikur R. M. Chowdhury M. R. Moniruzzaman M. Goto M. (2020). Biocompatible ionic liquids and their applications in pharmaceutics. Green Chem. 22 (23), 8116–8139. 10.1039/d0gc02387f

[B35] Moshikur R. M. Goto M. (2021). “Ionic liquids as active pharmaceutical ingredients (APIs),” in Application of ionic liquids in drug delivery. Editors Goto M. Moniruzzaman M. (Singapore: Springer Singapore), 13–33.

[B36] Musiał M. Zorębski E. Malarz K. Kuczak M. Mrozek-Wilczkiewicz A. Jacquemin J. (2021). Cytotoxicity of ionic liquids on normal human dermal fibroblasts in the context of their present and future applications. ACS Sustain. Chem. Eng. 9 (22), 7649–7657. 10.1021/acssuschemeng.1c02277

[B37] Nabila F. H. Islam R. Shimul I. M. Moniruzzaman M. Wakabayashi R. Kamiya N. (2024). Ionic liquid-mediated ethosome for transdermal delivery of insulin. Chem. Commun. 60 (30), 4036–4039. 10.1039/d3cc06130b 38466016

[B38] Navti P. D. Pandey A. Nikam A. N. Padya B. S. Kalthur G. Koteshwara K. B. (2022). Ionic liquids assisted topical drug delivery for permeation enhancement: formulation strategies, biomedical applications, and toxicological perspective. AAPS PharmSciTech 23 (5), 161. 10.1208/s12249-022-02313-w 35676441

[B39] Niesyto K. Keihankhadiv S. Mazur A. Mielańczyk A. Neugebauer D. (2024a). Ionic liquid-based polymer matrices for single and dual drug delivery: impact of structural topology on characteristics and *in vitro* delivery efficiency. Int. J. Mol. Sci. 25 (2), 1292. 10.3390/ijms25021292 38279291 PMC10816880

[B40] Niesyto K. Mazur A. Neugebauer D. (2024b). Piperacillin/tazobactam Co-delivery by micellar ionic conjugate systems carrying pharmaceutical anions and encapsulated drug. Pharmaceutics 16 (2), 198. 10.3390/pharmaceutics16020198 38399252 PMC10891911

[B41] Pansuriya R. Patel T. Kumar S. Aswal V. K. Raje N. Hoskins C. (2024). Multifunctional ionic hydrogel-based transdermal delivery of 5-fluorouracil for the breast cancer treatment. ACS Appl. Bio Mater. 7 (5), 3110–3123. 10.1021/acsabm.4c00152 38620030

[B42] Patil M. L. Sasai H. (2008). Recent developments on chiral ionic liquids: design, synthesis, and applications. Chem. Rec. 8 (2), 98–108. 10.1002/tcr.20143 18383110

[B43] Paz Ramos A. Gooris G. Bouwstra J. Molinari M. Lafleur M. (2020). Raman and AFM-IR chemical imaging of stratum corneum model membranes. Can. J. Chem. 98 (9), 495–501. 10.1139/cjc-2019-0471

[B44] Pham T. P. T. Cho C.-W. Yun Y.-S. (2010). Environmental fate and toxicity of ionic liquids: a review. Water Res. 44 (2), 352–372. 10.1016/j.watres.2009.09.030 19854462

[B45] Shukla M. K. Tiwari H. Verma R. Dong W. L. Azizov S. Kumar B. (2023). Role and recent advancements of ionic liquids in drug delivery systems. Pharmaceutics 15 (2), 702. 10.3390/pharmaceutics15020702 36840024 PMC9963759

[B46] Sidat Z. Marimuthu T. Kumar P. du Toit L. C. Kondiah P. P. D. Choonara Y. E. (2019). Ionic liquids as potential and synergistic permeation enhancers for transdermal drug delivery. Pharmaceutics 11 (2), 96. 10.3390/pharmaceutics11020096 30813375 PMC6409523

[B47] Stark A. Behrend P. Braun O. Müller A. Ranke J. Ondruschka B. (2008). Purity specification methods for ionic liquids. Green Chem. 10 (11), 1152–1161. 10.1039/b808532c

[B48] Tanner E. E. Ibsen K. N. Mitragotri S. (2018). Transdermal insulin delivery using choline-based ionic liquids (CAGE). J. Control. release 286, 137–144. 10.1016/j.jconrel.2018.07.029 30026081

[B49] Thuy P. T. P. Cho C.-W. Yun Y.-S. (2010). Environmental fate and toxicity of ionic liquids: a review. Water Res. 44 (2), 352–372. 10.1016/j.watres.2009.09.030 19854462

[B50] Uddin S. Islam M. R. Chowdhury M. R. Wakabayashi R. Kamiya N. Moniruzzaman M. (2021). Lipid-based ionic-liquid-mediated nanodispersions as biocompatible carriers for the enhanced transdermal delivery of a peptide drug. ACS Appl. bio Mater. 4 (8), 6256–6267. 10.1021/acsabm.1c00563 35006923

[B51] Wang C. Zhu J. Zhang D. Yang Y. Zheng L. Qu Y. (2018). Ionic liquid – microemulsions assisting in the transdermal delivery of Dencichine: preparation, *in-vitro* and *in-vivo* evaluations, and investigation of the permeation mechanism. Int. J. Pharm. 535 (1), 120–131. 10.1016/j.ijpharm.2017.10.024 29104058

[B52] Zhang F. Li L. Zhang X. Yang H. Fan Y. Zhang J. (2024). Ionic liquid transdermal patches of two active ingredients based on semi-ionic hydrogen bonding for rheumatoid arthritis treatment. Pharmaceutics 16 (4), 480. 10.3390/pharmaceutics16040480 38675141 PMC11053956

[B53] Zhang S. Sun N. He X. Lu X. Zhang X. (2006). Physical properties of ionic liquids: database and evaluation. J. Phys. Chem. reference data 35 (4), 1475–1517. 10.1063/1.2204959

[B54] Zhuo Y. Cheng H.-L. Zhao Y.-G. Cui H.-R. (2024). Ionic liquids in pharmaceutical and biomedical applications: a review. Pharmaceutics 16 (1), 151. 10.3390/pharmaceutics16010151 38276519 PMC10818567

